# Short- and Long-Term Renal Outcome of Hemolytic-Uremic Syndrome in Childhood

**DOI:** 10.3389/fped.2018.00220

**Published:** 2018-08-07

**Authors:** Laura Vaterodt, Johannes Holle, Dieter Hüseman, Dominik Müller, Julia Thumfart

**Affiliations:** ^1^Department of Pediatric Gastroenterology, Nephrology and Metabolic Diseases, Charité – Universitätsmedizin Berlin, Berlin, Germany; ^2^Department of Pediatric Pneumology and Immunology, Charité – Universitätsmedizin Berlin, Berlin, Germany; ^3^Department of Pediatrics, Werner-Forßmann-Krankenhaus, Eberswalde, Germany

**Keywords:** hemolytic uremic syndrome, children, renal sequelae, EHEC, Shigatoxin

## Abstract

**Introduction:** Hemolytic-uremic syndrome (HUS) is a common cause for intrarenal acute kidney injury in childhood. More than 90% of HUS cases are associated with an infection by Shigatoxin-producing *Escherichia coli* (STEC) whereas the reminder comprises a heterogeneous group (here classified as Non-STEC-HUS). Renal impairment can persist in patients with HUS. This study presents data from four decades investigating the short- and long-term outcome of HUS in childhood.

**Materials and Methods:** In a retrospective single-center-study clinical and laboratory data of the acute phase and of 1- to 10-year follow-up visits of children with HUS were analyzed.

**Results:** 92 HUS-patients were identified from 1996 to 2014 (STEC-HUS-group: *n* = 76; Non-STEC-HUS-group: *n* = 16) and 220 HUS-patients between 1976 and 1995. STEC-HUS was increasingly caused by Non-O157 strains and mortality rate declined over the past decades (1.3 vs. 9.5%). Renal sequelae persisted more often in the group 1976–1995 (39.3%) than in the group 1996–2014 (28.3%), but more than 50% of all patients were lost to follow-up.

**Conclusion:** Although renal outcome has improved over the investigated last decades, patients with HUS still face a high risk of permanent renal damage. These findings underline the importance of a consequent long-term follow-up in HUS-patients.

## Introduction

Hemolytic-uremic syndrome (HUS) is defined by a triad of microangiopathic hemolytic anemia, thrombocytopenia and acute kidney injury and belongs to the heterogeneous group of thrombotic microangiopathy (TMA) (1–3).

HUS is a rare disease with the highest incidence in young childhood (6/100,000 children <5 years) in Western Europe and USA (2, 4), where it is a common cause for intrarenal acute kidney injury (AKI) (5). Most cases occur sporadically, but epidemic outbreaks are seen from time to time (e.g., Germany, 2011) (6).

Traditionally, HUS was classified by clinical presentation in prodromal phase as Diarrhea-positive (D^+^) or typical HUS and Diarrhea-negative (D^−^) or atypical HUS. The former primarily resulted from Shigatoxin-producing *Escherichia coli* (STEC) infections and accounts for more than 90% of HUS cases in childhood and adolescence (7). All other cases were summarized as atypical, Diarrhea-negative HUS, even though some of these patients also presented with diarrhea.

A novel etiology-based classification has evolved (8), which was recently adapted to our national guideline^1^ as

1. STEC-HUS (formerly typical or Diarrhea-associated HUS)
2. Pneumococcus associated HUS
3. Complement-mediated HUS
4. Other types of HUS (e.g., Cobalamin-C- or DGKE-mutations, drugs, medication, systemic lupus erythematosus or pregnancy)

STEC-HUS is usually caused by an infection with STEC or, less frequent, *Shigella dysenteriae* Type 1. Typically, affected children are between 2 and 5 years of age. Patients with STEC-HUS present with diarrhea, often bloody, in the prodromal phase. Renal impairment occurs usually 7–14 days after infection. Extrarenal complications occur more frequently in central nervous system, but are also seen in pancreas, gastrointestinal tract and cardiorespiratory system (4, 9, 10).

In most cases, the diagnosis of STEC-HUS can be made by (typical) medical history, clinical presentation and (basic) laboratory diagnostics. In patients with STEC-HUS or Pneumococcus associated HUS a pathogen detection (blood and/or stool sample) should be obtained. In patients with complement-mediated HUS detailed complement diagnostics, including genetic analysis, in specialized laboratories are necessary.

Therapy during the acute phase is basically supportive. 50–70% of patients with HUS need renal replacement therapy and up to 80% require transfusions (red blood cells, platelets) (5, 11, 12). Eculizumab, a monoclonal C5 antibody, significantly improved renal outcome and survival of patients with complement-mediated HUS (13, 14).

Mortality in the acute phase is low (<5%) (15), but renal impairment persists in up to 30% of patients with STEC-HUS (12, 16).

In this single-center-study we investigated data of our HUS-patients of four decades with the aim to improve the understanding of short- and long-term outcome of HUS in childhood and identifying prognostic markers at first presentation.

## Patients and methods

In a retrospective single-center-study we investigated clinical and laboratory data out of the acute phase and the 1- to 10-year follow-up visits in children diagnosed with HUS between the years 1976 and 2014.

Patients were divided in two groups, according to time of diagnosis and etiology.

Patients with onset between 1996 and 2014 were assigned to either STEC-HUS- or Non-STEC-HUS-group (group 2). Detection of EHEC/STEC or Shigatoxin (stx) in stool or blood sample or typical clinical presentation with (bloody) diarrhea, age of onset 2–5 years and neither relapse nor familiarity attributed patients to STEC-HUS-group. All other patients were included as Non-STEC-HUS-group.

All patients with disease onset between 1976 and 1995 were summarized as group 1 or a historical cohort. In this group, differentiation between STEC- and Non-STEC-HUS was not feasible due to insufficient/non-existent data on EHEC/stx detection and complement diagnostics.

We investigated demographic (gender, age, body height, and weight), clinical (symptoms, arterial blood pressure and extrarenal complications) and laboratory data (hemoglobin, platelet count, leucocyte count, serum creatinine (Jaffe or enzymatic method), estimated glomerular filtration rate (eGFR), urinary output, complement factor C3, proteinuria) and medication (antihypertensive drugs, renal replacement therapy, transfusions) on admission and during the acute phase. Follow-up parameters (arterial blood pressure, need for antihypertensive medication, eGFR and proteinuria) were investigated at onset and thereafter once a year until transition to adult medical care. eGFR was calculated by updated Schwartz' formula and, if available in early cases, results of the isotope clearance method were also collected (17, 18).

Blood pressure was measured with an appropriately sized cuff either by oscillometric devices or by auscultation. Because of insufficient data (height and weight) in follow-up visits age- and gender-adjusted percentiles derived from 24 h ambulatory blood pressure monitoring were applied (19, 20).

Surrogates for possible kidney injury were defined as the presence of at least one of the following parameters: proteinuria ≥300 mg/l or ≥1+ on dipstick, mean arterial pressure (MAP) >90th Percentile (adjusted for age and sex) (20), need for antihypertensive medication, and eGFR <90 ml/min/1.73 m^2^. Renal sequelae were defined as need for antihypertensive medication and/or eGFR <90 ml/min/1.73 m^2^.

### Statistics

SPSS Version 22 (IBM, 2013) and Microsoft Excel (2013) were used for data collection and statistical analysis. Normality of variables was tested by Kolmogorov–Smirnov Test. As for bivariate statistics, the dependence between the data was tested using Fisher's exact test (small sample size, frequency <5) or chi^2^ test for count data. Mann–Whitney *U*-test was used to compare continuous variables. *P* < 0.05 were considered statistically significant.

### Ethical approval

All procedures performed in studies involving human participants were in accordance with the ethical standards of the institutional and national research committee and with the 1964 Helsinki declaration and its later amendments.

## Results

### Study collective

**Figure 1:**
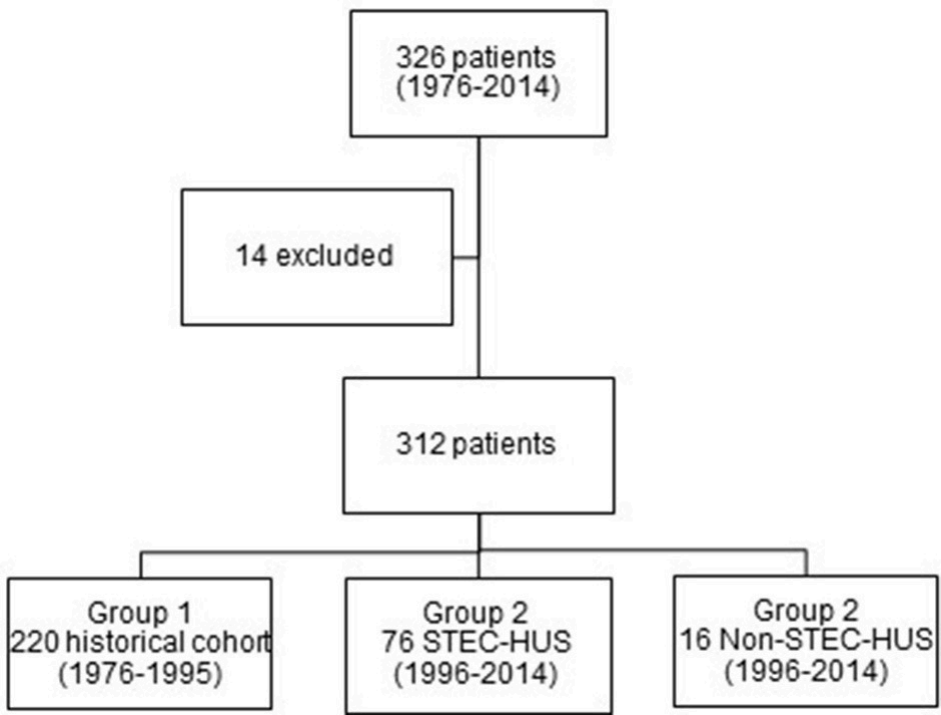
Study collective.

In total, 326 children with the diagnosis of HUS have been identified between 1976 and 2014. 14 patients (4.3%) were excluded due to insufficient data (Figure 1). There was an almost similar frequency of females (52.6%) and males (47.4%).

Most of the patients were treated in the time period between 1976 and 1995 (220 patients, 70.5%). In the second period, 76 patients with STEC-HUS and 16 patients with Non-STEC-HUS-group were treated in our hospital (Figure 1).

### Epidemiology and etiology

#### Group 1 (1976–1995, n = 220)

We identified 220 children with the diagnosis of HUS between 1976 and 1995 (115 females, 105 males). Their median age was 1.88 years [0.3; 17.0].

#### Group 2 STEC-HUS-GRoup (1996–2014, n = 76)

**Figure 2:**
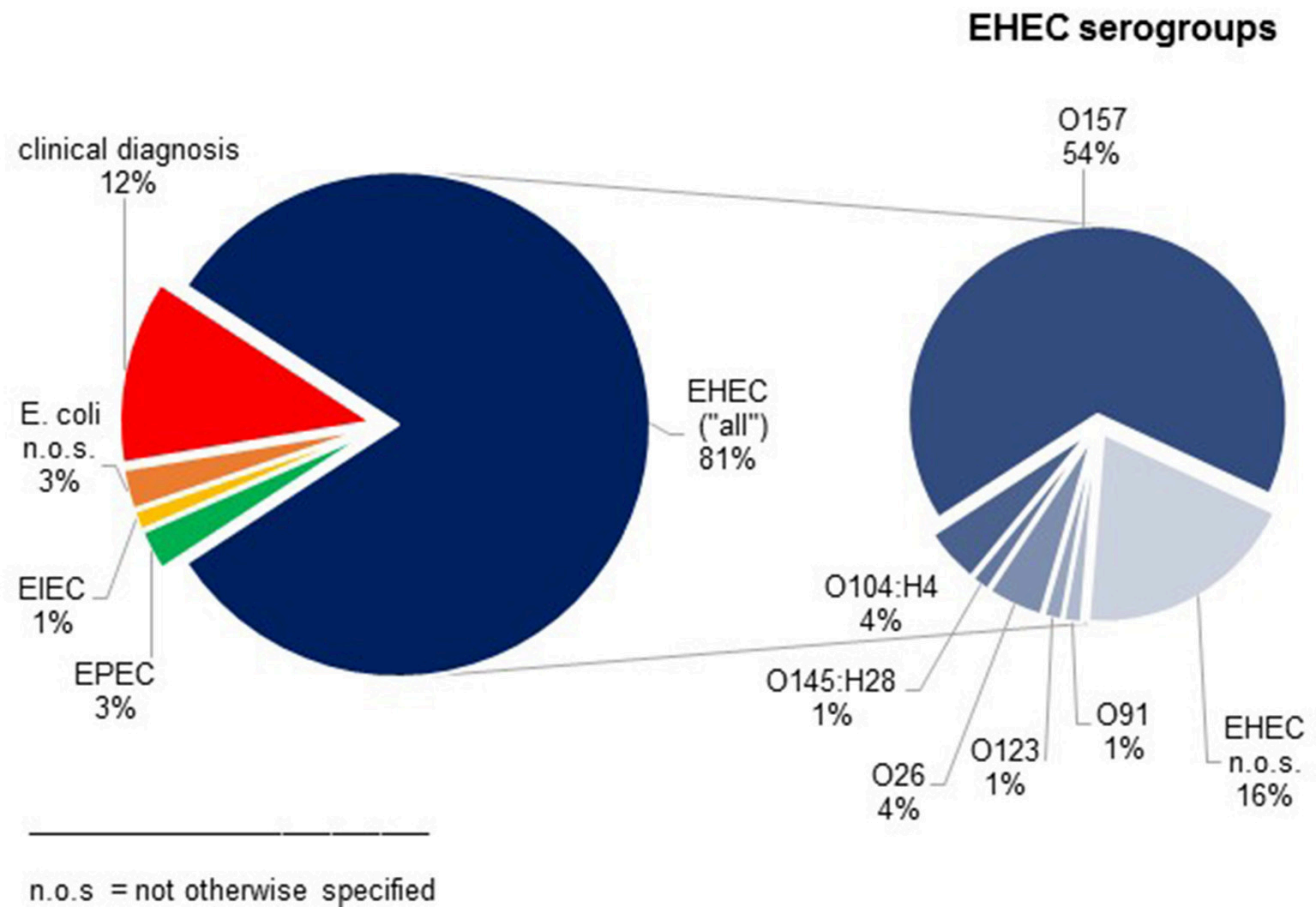
Results of microbiological analysis in patients with STEC-HUS. In 12% clinical diagnosis of STEC-HUS was suspected without detection of *E. coli*.

76 children (44 girls, 32 boys) with STEC-HUS were identified between 1996 and 2014. Their median age was 3.0 years [0.3; 15.7]. Most cases occurred between the months May and September. Clinical HUS diagnosis was confirmed by microbiological analysis in 88% of the patients. Most frequently detected pathogen was EHEC O157 (41/76 STEC-HUS-patients, 54%) (Figure 2). Shigatoxin was detected in 73% (40/55), especially stx 2 in 63% (35/50).

#### Group 2 Non-STEC-HUS-Group (1996–2014, n = 16)

We identified 16 patients (five females, 11 males, median age 2.4 years, [0.4; 20.0]) with Non-STEC-HUS from 1996 to 2014. Patients with Non-STEC-HUS were observed during all seasons. Different etiologies for Non-STEC-HUS were observed, most common was Pneumococcus associated HUS and complement-mediated HUS (Table 1). Five patients were classified into the Non-STEC HUS group despite diarrhea. These patients had Pneumococcal HUS (*n* = 1), Aeromonas associated HUS (*n* = 1) and complement-mediated HUS (*n* = 3). Complement-mediated, familial HUS was diagnosed in two cases: one boy had two episodes of HUS without signs of infections. His older brother had also recurrent HUS episodes. The other boy had also HUS without signs of infection. His mother and grandmother had had recurrent HUS episodes.

**Table 1:** Cohort of patients with Non-STEC-HUS.

Post-infectious	**6**
- *S. pneumoniae*	5
- *Aeromonas* spp.	1
Complement-mediated	**5**
- Factor-H-antibodies + CFHR-deficiency	3
- Familial	2
MMACHC^†^-mutation	**1**
Bone marrow transplantation	**1**
Etiology unknown	**3**

† *MMACHC, methylmalonic aciduria and homocystinuria type C protein. Bold values represent the sum of each category*.

The three patients, which we classified as unknown etiology (Table 1), had no reported diarrhea, low C3 serum and negative results for EHEC diagnostic.

### Laboratory data, clinical presentation and therapy (acute phase)

Most patients with STEC-HUS presented with a prodromal phase of 5 days with diarrhea (90.5%), often bloody, vomiting and fever. These symptoms were seen less frequently in patients with Non-STEC-HUS (diarrhea 33.3%). Median duration of the prodromal phase in patients with Non-STEC-HUS was 3 days, clinical presentation varied.

Acute renal failure was similar in both groups [STEC-HUS 44/62 (71.0%), Non-STEC-HUS 10/13 (76.9%)], but the need of antihypertensive medication was higher in Non-STEC-HUS-group (35.1% vs. 56.3%). Extrarenal manifestations were encountered more frequently in Non-STEC-HUS-group (Table 2). Decrease in complement factor C3 was seen in 19% of STEC-HUS- and 50% of Non-STEC-HUS-group.

**Table 2:** Laboratory data, clinical presentation, therapy and mortality rate during acute phase (STEC- and Non-STEC-HUS-group, 1996–2014).

	**STEC-HUS-group (1996–2014**, ***n*** = **76)**		**Non-STEC-HUS-group (1996–2014**, ***n*** = **16)**		***p***
**Laboratory data**					
	median (*n*) [range]		median (*n*) [range]		
- Hemoglobin (admission)	8.8 g/dl (72) [3.9–15.5]		8.7 g/dl (16) [4.3–14.6]		
- White blood cells (admission)	15/nl (73) [4.2–43.0]		11/nl (16) [4.8–20.2]		
- Platelets (min)	44/nl (73) [9.0–414.0]		21/nl (15) [8.0–142.0]		
- Se-creatinine (max)	4.6 mg/dl (73) [0.34–11.6]		3.7 mg/dl (14) [1.0–9.1]		
- Complement factor C3 ↓	11/58 = 19%		5/10 = 50%		0.033
**Clinical presentation**					
	(“yes”/*n*)	%	(“yes”/*n*)	%	
Diarrhea	67/74	90.5	5/15	33.3	0.000
- Bloody	28/74	37.8			
Fever	15/74	20.3	5/15	33.3	0.419
Renal manifestation					
- Olig-/anuria	44/62	71.0	10/13	76.9	0.664
- Anuria > 7 days	9		7		
- Antihypertensive medication	26/74	35.1	9/16	56.3	0.116
Extrarenal manifestation					
- Central nervous system	18/75	24.3	6/16	37.5	0.266
- Pancreas	4/75	5.3	1/15	6.7	0.837
**Therapy**					
	(“yes”/*n*)	%	(“yes”/*n*)	%	
Dialysis	59/76	77.6	13/16	81.3	0.750
- HD^†^	6		4		
- PD^‡^	45		6		
- PD and HD	8		3		
Plasmapheresis	9/76	11.8	4/16	25.0	0.170
Eculizumab	0		1		
Antihypertensive medication	26/74	35.1	9/16	56.3	0.116
Transfusions					
- Red blood cells	63/75	84.0	9/16	56.3	0.013
- Platelets	8/74	10.8	5/16	31.3	0.035
- Fresh frozen plasma	5/74	6.8	5/16	31.3	0.005
**Mortality rate** (acute phase)	1/76	1.3	1/16	6.3	0.219
**Renal transplantation**	5/75	6.7	5/16	31.3	0.004

† HD, hemodialysis;

‡ *PD, peritoneal dialysis; ↓Decreased complement C3 level*.

59 of 76 children with STEC-HUS (77.6%) and 13 of 16 patients with Non-STEC-HUS (81.3%) needed renal replacement therapy. Plasmapheresis was performed in nine STEC-HUS-patients (11.8%) and four Non-STEC-HUS-patients (25.0%). One patient with Non-STEC-HUS received Eculizumab (Table 2).

75 of 76 (98.7%) patients with STEC-HUS and 15 of 16 (92.8%) Non-STEC-HUS-patients survived the acute phase. Compared to the historical group (group 1), mortality rate in the acute phase was significantly lower (1.3 vs. 9.5%, *p* = 0.018) in the STEC-HUS-group.

### Long-term follow-up

**Figure 3:**
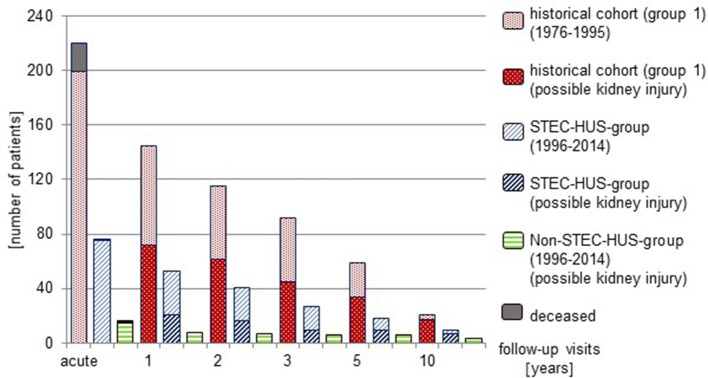
Long-term follow-up and potential renal damage (1976–2014).

It was recommended to all patients with HUS to adhere to regular follow-up visits. However, a high proportion of patients was lost to follow-up (Figure 3).

At 1 year follow-up, 53 of 75 (70.6%) patients with STEC-HUS were seen in the outpatient ward. 21 of these patients (39.6%) were identified to have surrogates for possible kidney injury. Renal sequelae showed 15 patients (28%). 8 of 15 (53.3%) patients with Non-STEC-HUS were seen at follow-up and all of these had renal sequelae. In the historical group, 145 of 199 patients (72.8%) were seen and 72 (49.7%) had surrogates for possible kidney injury. 57 (39.3%) presented with renal sequelae.

At 5 year follow-up, 9/18 STEC-HUS-patients (50.0%) had surrogates for possible kidney injury; 8 (44.4%) had renal sequelae. 6/6 Non-STEC-HUS-patients seen in the outpatient ward had renal sequelae. In the historical cohort 34/59 of patients (57.6%) who were seen after 5 years showed surrogates for renal damage and 24 (40.7%) had renal sequelae.

At 10 year follow-up, 7/9 STEC-HUS-patients (77.8%) and 3/3 Non-STEC-HUS-patients who came to follow-up showed renal sequelae. 17/21 patients from the historical cohort (81.0%) had surrogates for possible kidney injury, 15 of these (71.4%) had renal sequelae.

Rate of renal transplantations was significantly higher in patients with Non-STEC-HUS (31.3%) in comparison to STEC-HUS-group (6.7%, *p* = 0.004) (Table 2).

### Risk-factors for potential renal damage

We found the strongest correlation between antihypertensive therapy during the acute phase as well as anuria >7 days and surrogates for possible kidney injury in 1- to 5-year follow-up (STEC-HUS-group and historical cohort, Table 3). No significant correlations (*p* ≥ 0.05) were found for platelet count <50/nl or GFR <20 ml/min/1.73 m^2^ on admission as well as age or sex (STEC-HUS-group and historical cohort). Extrarenal involvement (central nervous system/pancreas) was not a significant risk-factor for surrogates for possible kidney injury (*p* ≥ 0.05) in 1- to 5-year follow-up. In the STEC-HUS-group, 18 patients had central nervous system involvement and four patients pancreatic affection. Out of these patients 10 had surrogates for possible kidney injury, seven showed no signs of renal damage and five patients were lost to follow up. Decrease of complement factor C3 and proof of Shigatoxin 2 (stx 2) were not associated with surrogates for possible kidney injury in the STEC-HUS-group (1996–2014).

**Table 3:** Risk factors for potential renal residual damage.

**Acute phase**	**1-year follow-up**		**2-year follow-up**		**3-year follow-up**		**5-year follow-up**	
Groups	hist.^†^	STEC	hist.	STEC	hist.	STEC	hist.	STEC
AHT^§^	0.003	0.003	n.s.^¶^	<0.0001	0.022	0.023	0.029	0.029
Anuria >7 days	<0.0001	0.038	0.01	0.028	0.004	n.s.	0.025	n.s.
Leukocytes >20/nl	0.01	n.s.	0.02	n.s.	n.s.	0.007	0.006	n.s.
Dialysis	n.s.	0.002	n.s.	0.031	n.s.	n.s.	n.s.	n.s.

*Corresponding p-values are shown*.

† hist., historical cohort (1976–1995, group 1); STEC, STEC-HUS-group (1996–2014);

§ AHT, antihypertensive therapy;

¶ *n.s., not significant (p ≥ 0.05)*.

## Discussion

In this study we report on 312 children, which is one of the largest HUS cohorts published. We analyzed epidemiological, clinical and laboratory data as well as follow-up data up to 10 years. One of the challenges, reporting about HUS in the past decades, is how to consider the changes in pathophysiology and disease classification. Primarily, HUS classification was orientated by clinical manifestations (D^+^/D^−^ HUS, typical/atypical HUS). In contrast, in the recent years, a more detailed classification was established (8). Therefore, we decided to report about a historical cohort (group 1) between 1976 and 1995, where differentiation between D^+^/D^−^ or typical/atypical HUS would be speculative, and a cohort between 1996 and 2014 (group 2). With regard to the current knowledge in pathophysiology we sorted the patients into a STEC-HUS- or Non-STEC-HUS-group.

In STEC-HUS-group females and males were affected equally and age of onset was similar to Non-STEC-HUS-group. STEC-HUS most commonly occurred during the summer season whereas Non-STEC-HUS cases were observed all over the year. This data confirms other cohort studies (21–23). EHEC O157 still was the most common strain in STEC-HUS-group, but as observed in other national and international cohorts, Non-O157 strains become more prevalent (21, 24, 25).

In the acute phase of illness, as shown in Table 2, neither laboratory nor clinical data allowed an absolute discrimination between STEC-HUS and Non-STEC-HUS. In our cohort, one third of Non-STEC-HUS-patients presented with diarrhea and fever, on the other hand almost 20% of STEC-HUS-patients showed signs of complement activation (decrease of serum C3). In the Non-STEC-HUS group might be cases of STEC-HUS. Missing detection of STEC does not exclude STEC-HUS (26). However, in our Non-STEC-HUS group are only three patients with unknown etiology. These patients had low serum C3 levels, no reported diarrhea and negative results for EHEC diagnostic.

Differentiation between STEC- and Non-STEC-HUS is further complicated by the fact that stx can trigger complement-dependent microvascular thrombosis (27). These observations might justify the use of eculizumab in severe cases of STEC-HUS with neurological disorders (28).

Compared to other recently published cohort studies (21–23), the frequency of renal replacement therapy was higher in our patients with almost 80%. Additionally frequency of patients with HUS was declining in our hospital over the past decades. One reason for these aspects might be that more patients without need of renal replacement therapy are treated in other hospitals.

The need of antihypertensive medication was higher in Non-STEC-HUS-group than in STEC-HUS-group.

Mortality rate was low for STEC-HUS (1.3%) and Non-STEC-HUS-group (6.3%) and declined compared to historical group (9.5%) as better treatment options, especially for Non-STEC-HUS, are available. Compared to other cohorts mortality rate was comparable low (15, 21, 22).

Renal impairment persists in 25–30% of patients with EHEC/Diarrhea associated HUS (12, 16). Elevated white blood cell count at the beginning of the illness, antihypertensive therapy during acute phase and extrarenal involvement (particularly central nervous system and pancreas) are described as risk-factors for renal sequelae. Furthermore, the duration of olig-/anuria, as well as need for renal replacement therapy may be associated to a worse renal long-term outcome (12, 16, 29, 30). In our study, we confirmed that anuria for more than 7 days and the need of antihypertensive therapy in the acute phase of illness strongly correlate with surrogates for possible kidney injury after 1–5 years (STEC-HUS-group and historical cohort). In contrast, platelet count <50/nl, GFR <20 ml/min/1.73 m^2^ or extrarenal involvement were no risk-factors for surrogates for possible kidney injury in our study collective.

We saw a steady lost to follow-up over the years, meanwhile proportion of patients with renal damage who came to follow-up was increasing through the years. There might be some selection bias over time. However, in our study, almost one third of children with possible kidney injury are lost to follow up. Nearly one sixth of children who were classified with no signs of renal damage in the first year follow-up visit showed surrogates for possible kidney injury 1 year later on.

Low follow-up rates were also seen in other cohort studies (21, 22).

It seems to be very difficult to reach adequate follow-up rate, even in a single center. Achieving improvement here seems to be very important, as a high proportion of our patients has persistent renal sequelae.

There are several limitations in our study. First, we performed a retrospective single-center-study and therefore missed the advantages of prospective multicenter trials. Further, data collection was incomplete in some cases. As our study covered almost four decades, diagnostic tests and classifications changed over this period, so we had to work with several compromises in describing our patient cohort. Our study was monocentric and as children without complete renal failure (and without need of renal replacement therapy) may have been treated in other hospitals, we have some bias here regarding incidence rate and severity of clinical course. As in other cohort studies, we have low follow-up rates and therefore can only speculate about real incidence of renal sequelae after HUS. Finally, we did not register data about the severity of renal sequelae in detail.

In conclusion, our report points at the fact that all patients, irrespective of their HUS origin, strictly should be followed on a long-term basis because of the high impact of renal sequelae for their further life. In order to reach an improvement here, national or international guidelines should be implemented with detailed information and instruction for consequent follow-up in all patients. Further, national or international registries could be implemented in order to measure and improve renal outcome after HUS.

## Author contributions

All authors (LV, JH, DH, DM, and JT) collected the clinical data, analyzed the data and participated in wrinting the manuscript.

### Conflict of interest statement

The authors declare that the research was conducted in the absence of any commercial or financial relationships that could be construed as a potential conflict of interest.

## Footnotes

^1^*AWMF: Detail*. Available online at: http://www.awmf.org/leitlinien/detail/ll/166-002.html (Accessed April 28, 2018).
